# Resonances in Low-Energy Electron Collisions with Salicylic Acid

**DOI:** 10.1021/acs.jpca.4c07822

**Published:** 2025-01-28

**Authors:** Valéria Liberti, Pedro A. S. Randi, Márcio H. F. Bettega, Alessandra Souza Barbosa

**Affiliations:** Departamento de Física, Universidade Federal do Paraná, Caixa Postal 19044, 81531-980 Curitiba, Paraná, Brazil

## Abstract

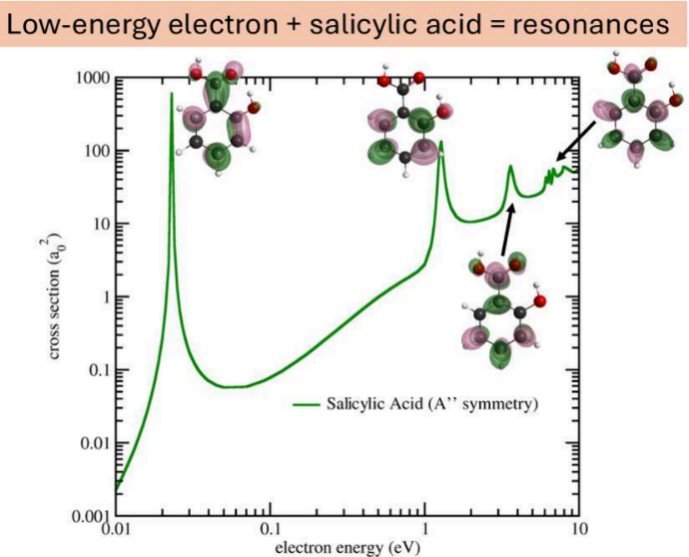

In this work, we report elastic integral, differential, and momentum-transfer cross sections for the scattering of low-energy electrons by salicylic acid. The cross sections were calculated with the Schwinger multichannel method implemented with norm-conserving pseudopotential within the static-exchange and static-exchange plus polarization (SEP) approximations for energies up to 15 eV. In the SEP approximation, four π* resonances were found at around 0.023, 1.27, 3.60, and 6.80 eV. While the first three are shape resonances, the latter has a mixed shape and core-excited shape character. We compare our results with available measurements in the literature, and we also discuss the role of the second resonance in the production of [M-H]^−^ and [M-H_2_]^−^ species through dissociative electron attachment.

## Introduction

Since the seminal work of Boudaïffa et al.,^1^ the interest of the scientific community in the interaction between low-energy electrons and biologically relevant molecules has grown. These authors showed that low-energy electrons are capable of damaging the genetic material by inducing single- and double-strand breaks of DNA molecules. This damage is driven by dissociative electron attachment (DEA) to one of DNA’s nucleobases. In this process, the incident electron is temporarily captured through a resonant process by one of the nucleobases of DNA. This resonant state can either decay via autodetachment or lead to molecular dissociation, the latter resulting in damage to the DNA molecule itself. Following this work, significant efforts have been made to thoroughly understand the interactions between low-energy electrons and biological molecules (see, for instance, Baccarelli et al.,^2^ Gorfinkiel and Ptasinska^3^ and references therein).

**Figure 1:**
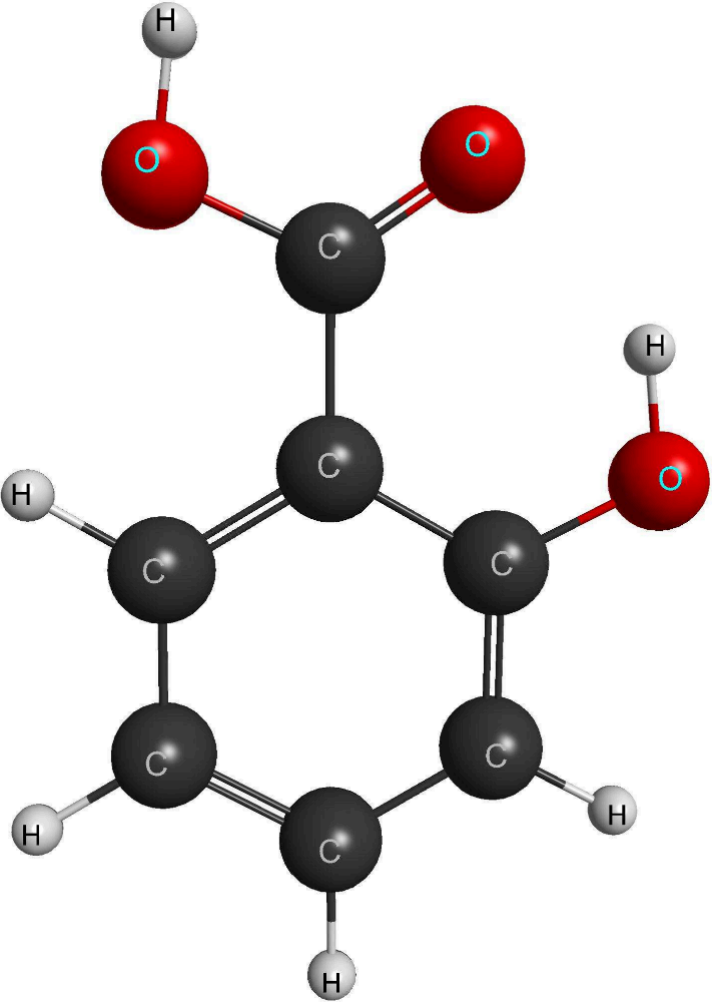
Ball and stick model of salicylic acid (generated with MACMOLPLT^4^).

Among relevant building blocks of biological molecules, salicylic acid (SA, Figure 1) is the primary active ingredient in the salicylate family, a class of nonsteroidal anti-inflammatory drugs with analgesic and antipyretic properties. The name salicylic acid comes from the Latin word *Salix*, which refers to the willow tree, and in the 19th century other salicylates were isolated from various plants and used medicinally.^5^ More specifically, in 1898, acetylsalicylic acid, known as aspirin, was introduced into the pharmaceutical industry and became one of the best-selling drugs in the world.^5^ Additionally, SA is associated with heat production in thermogenic plants and flowering and it signals the activation of disease resistance following pathogen infection in plants.^6^

Although the interaction between electrons and SA is relevant, to the best of our knowledge only two works have been found in the literature. In 2014, Scheer et al.^7^ reported the energies of four π* resonances employing electron transmission spectroscopy (ETS) and electronic structure calculations. Their calculations also suggest the existence of a σ*(OH) resonance not identified in their measurements. Pshenichnyuk and Modelli^8^ also investigated the resonances for this system through electron transmission spectroscopy, as well as the fragments produced by DEA using a dissociative electron attachment spectroscopy technique, relating these fragments to biochemical processes. As is evident, there is a lack of electron scattering cross sections for this molecule in the literature.

Here, we intend to further investigate the nature of the shape resonances of salicylic acid and provide the first set of reliable elastic electron scattering cross sections for this molecule. The integral (ICS), differential (DCS) and momentum-transfer (MTCS) elastic scattering cross sections for salicylic acid reported here were obtained employing state-of-art *ab initio* scattering calculations. These calculations were performed with the Schwinger multichannel method (SMC)^9,10^ implemented with norm-conserving pseudopotentials^11^ for energies up to 15 eV.

The rest of this paper is organized as follows: in the next section the main aspects of the SMC method and the computational details used in the present calculations are going to be described. Then, the results will be discussed, and a summary of our findings may be found in final section of this paper.

## Computational Details

All calculations were performed within the Born–Oppenheimer (fixed-nuclei) approximation in the optimized geometry of the most stable conformer of the molecule,^8^ depicted in Figure 1. This geometry has been optimized at the Hartree–Fock level, with the 6-31G(*d*) basis set, using the computational package GAMESS.^12^ Subsequent scattering calculations were carried out with the SMC method^9,10^ implemented with norm-conserving pseudopotentials of Bachelet, Hamann, and Schlüter (BHS).^11,13^ The SMC method and its implementations have recently been revised,^14^ thus only the key aspects of the present calculations will be discussed here.

In the SMC method, the scattering amplitude has a closed form written as
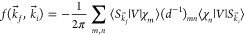
1where
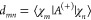
2

In the equations above, *V* is the interaction potential between the incident electron and the molecular target, {|χ_*m*_⟩} are (*N*+1)-electron trial configuration-state functions (CSFs) which are going to be discussed latter,  are the incident (scattered) electron wave vectors, and  is the solution of the unperturbed Hamiltonian (*H*_0_), being the product of a target state and a plane wave. The operator *A*^(+)^ in eq 2 is given by
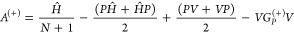
3where  is the total collision energy minus the (*N*+1)-electron Hamiltonian in the fixed nuclei approximation (*H* = *H*_0_ + *V*), *P* is a projector into the open elastic channel and  is the free particle Green’s function  projected onto the *P* space.

There are two ways in which the configuration space was constructed for the scattering calculations. In the first one, the CSFs are obtained as
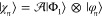
4where |Φ_1_⟩ is the Hartree–Fock target ground state, |φ_*n*_⟩ is a scattering orbital and  is the antisymmetrization operator. In this approximation, known as static-exchange (SE) approximation, the target electronic cloud is frozen in the ground state, such that polarization effects are completely omitted. Although the lack of polarization effects hinders the description of the low-energy scattering (impact energies typically  10 eV), where these effects are most important, the calculation performed in this approximation is relatively inexpensive, and qualitative features of the scattering process can be inferred, such as the formation of shape resonances.

The second way that we are able to construct the configuration space is through
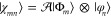
5where now |Φ_*m*_⟩ are *N*-electron Slater determinants obtained by performing single (virtual) excitations of the target, where an electron from an occupied (hole) orbital is excited to an unoccupied (particle) orbital. These single excitations introduce the polarization effects in the scattering calculations (single excitations of the target imply in double excitations of the negative ion), improving the description of the scattering process in the low-energy regime in relation to the SE approximation at the expanse of a more computationally costly calculation. This second calculation level is known as static-exchange plus polarization (SEP) approximation.

In the bound state and scattering calculations the nuclei and core electrons are described through the BHS pseudopotentials^13^ whereas the 5*s*, 5*p*, and 2*d* Cartesian Gaussian (CG) basis set describe the valence electrons. These CG functions were generated according to Bettega et al.,^15^ and the exponents of the functions were already employed in previous SMC calculations.^16^ For the hydrogen atoms, the 4*s*/3*s* Dunning basis set^17^ with an additional *p* type function with exponent 1.0 was used. In the SE approximation, we have used all unoccupied molecular orbitals as scattering orbitals in order to construct the CSFs. Meanwhile, for the CSFs of the SEP approximation, we chose the more compact modified virtual orbitals (MVOs)^18^ which were generated from a Fock operator with charge +4, preserving the same spatial and spin symmetries of the ground state. In particular, this set of MVOs provides low-lying valence type *a*″ virtual orbitals, relevant for the description of the shape resonances. To construct the CSFs we have used all 26 valence electrons as hole orbitals, while the first 31 MVOs were used as particle and scattering orbitals. This resulted in 13300 CSFs of the *A*′ symmetry and 12016 CSFs of *A*″ symmetry.

Since one of our goals in this work is to study the shape resonances of salicylic acid, we have performed additional analysis beyond the calculation of the cross sections themselves. Through the diagonalization of the scattering Hamiltonian (*H*_*N*+1_) in the SEP configuration space of each symmetry one can construct the Dyson orbitals following
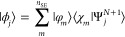
6where  are the eigenstates of *H*_*N*+1_, |φ_*m*_⟩ are scattering orbitals, |χ_*m*_⟩ are the CSFs and the sum runs over all *n*_SE_ CSFs that belong to the SE space (eq 4). These orbitals are proper representation of the resonant states, which aid in the qualitative characterization of the resonances.

The use of CG functions makes the integrals in eq 1 to be straightforwardly evaluated. However, since these functions are short-ranged, the long-range interactions between the incident electron and the permanent electric dipole of the molecule are poorly described by the SMC method. According to our calculation, the permanent dipole moment of salicylic acid is 2.39 D, in good agreement with others calculations.^19^ To overcome this limitation and provide accurate cross sections, we employed the well-known Born-closure procedure,^20^ following the same strategy described previously.^14^ In this procedure, the partial waves with higher angular momentum (above a chosen cut _SMC_ value) are calculated within the first Born approximation, while the partial waves with lower angular momentum are described by the SMC method. The _SMC_ is chosen such that the calculated corrected and uncorrected DCSs match for intermediate and higher scattering angles. The _SMC_ for each energy regime used in the present calculation can be found in Table 1. It is worth noting that the Born-closure procedure does not affect the resonances’ position, only the magnitude of the cross sections.

**Table 1:** _SMC_ Values Used for the Born-Closure Procedure^a^

_SMC_	Energies	_SMC_	Energies
1	0.01 to 0.34	6	4.00 to 5.70
2	0.35 to 1.29	7	5.80 to 7.30
3	1.30 to 1.70	8	7.40 to 13.0
4	1.80 to 2.00	9	14.0 to 15.0
5	2.10 to 3.90		

a Energies are in eV.

## Results and Discussion

**Figure 2:**
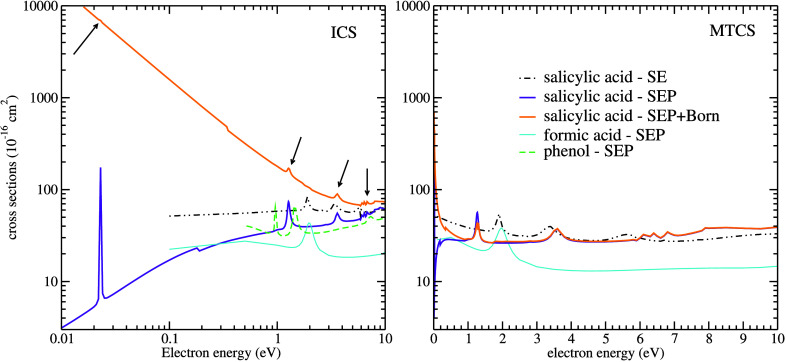
Integral (left panel) and momentum transfer (right panel) cross sections for elastic scattering of electrons by salicylic acid. SEP+Born approximation solid orange line, SEP approximation solid violet line, SE approximation dash-dot-dot black line, SEP approximation for phenol^23^ dashed green line and multichannel coupling approximation for formic acid^24^ solid cyan line. The arrows indicate the resonances in the SEP+Born ICS, which are masked by the Born-closure procedure.

Figure 2 shows the calculated ICS and MTCS, in left and right panels, respectively, in both the SE and SEP approximations and in the SEP + Born approximation, which accounts for the long-ranged electron-molecule interactions due to salicylic acid permanent electric dipole moment. The previous calculated cross sections of phenol^23^ and formic acid,^24^ obtained with the SMC method, are also presented in Figure 2 for comparison.

From Figure 2 it is noted some pronounced structures in both SE and SEP results. In particular, in the SE approximation, we can observe the formation of resonant structures at 1.9, 4.3, and 5.6 eV, and a broad structure at around 10 eV. Improving the description of the electron-molecule interaction by taking into account the distortion of the target electronic cloud in the presence of the incoming electron, in the SEP approximation, the resonances are shifted toward lower energies, being centered around 0.023, 1.27, 3.60, and 6.80 eV, respectively. In the SEP approximation, some sharp structures are seen at higher impact energies. These structures are pseudoresonances, which are nonphysical structures related to scattering channels that are energetically accessible, but treated as closed in the elastic approximation. When we perform the Born-closure procedure, the magnitude of the integral cross section increases dramatically in the low-energy regime while the position of the shape resonances remains unchanged, as expected.

**Figure 3:**
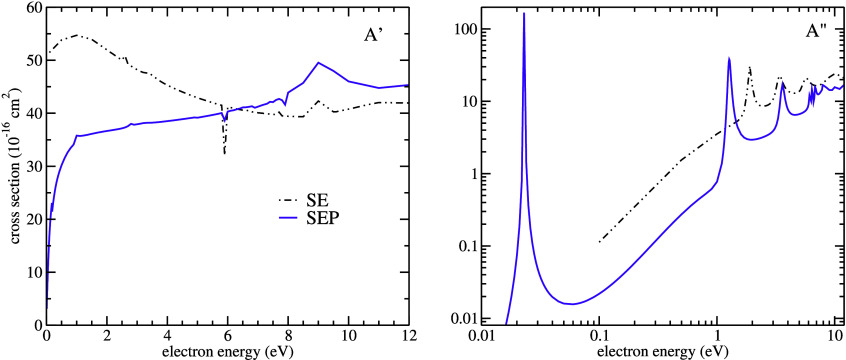
Symmetry decomposition of the ICS according to the *C*_*s*_ point group. SEP approximation solid violet line, SE approximation dash-dot-dot black line.

To help in the assignment of the resonant structures observed in the ICS, we present in Figure 3 the symmetry decomposition of the integral cross section, in the SE and SEP approximations, according to the *C*_*s*_ symmetry group. In the *A*′ symmetry, there is a broad structure at around 12 eV in the SE approximation, which moves to approximately 9 eV in the SEP approximation. It is also seen some narrow structures in the *A*′ symmetry which are due to numerical instabilities. In the *A*″ symmetry, we can see four well-defined structures, with the last one being slightly broader, in the SE approximation. For the SEP approximation, there are three well-defined peaks, which correspond to shape resonances, while the fourth one, at around 6 eV, presents some overlap with sharp structures known as pseudoresonances, suggesting that electronic excited states play a role at this energy and, thus, this may be a mixture of shape and core-excited shape resonances. This issue will be discussed further.

**Figure 4:**
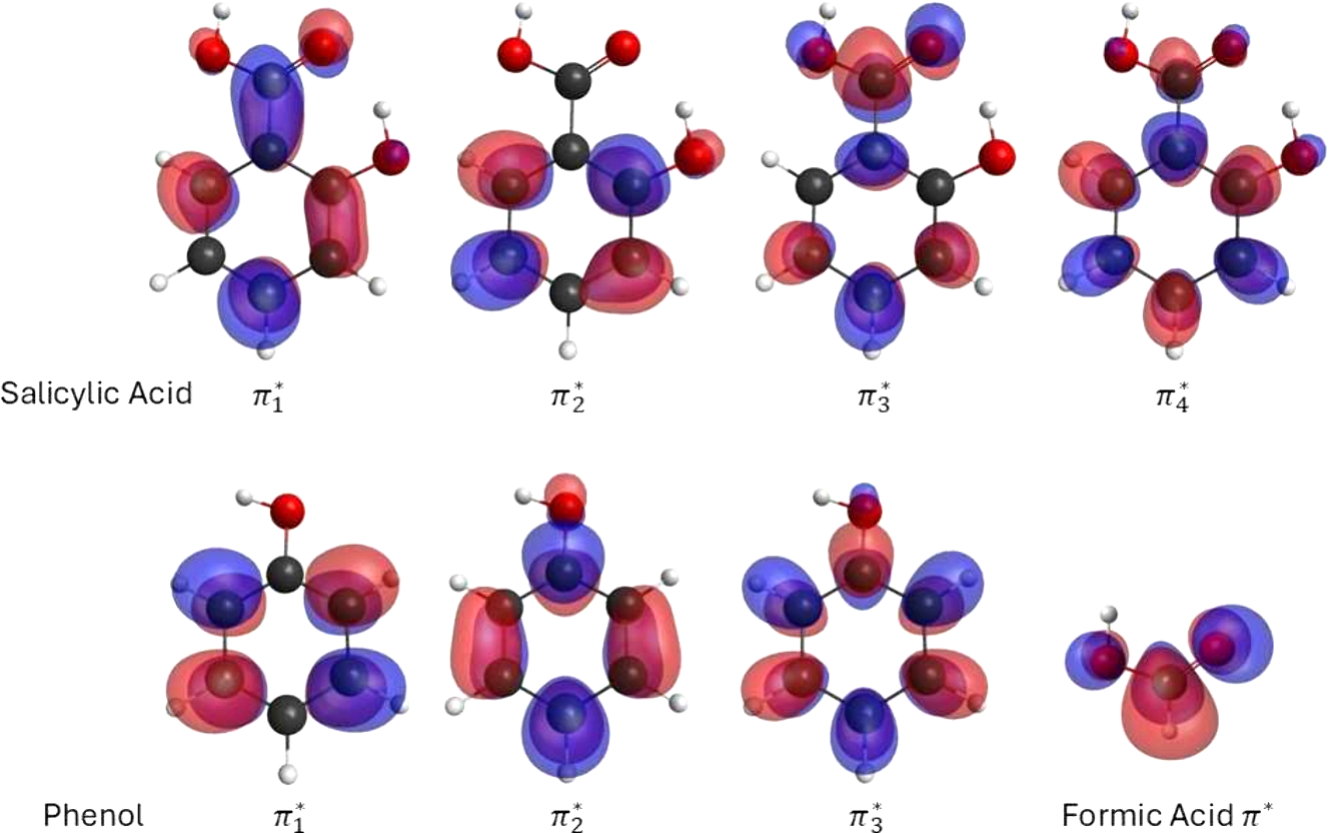
Four lowest π* resonant orbitals of salicylic acid (top). In the bottom is shown the three resonant π* resonant orbitals of phenol and the π* resonant orbital of formic acid.

From the literature, we have theoretical results obtained with the SMC method for phenol^22,23^ and formic acid,^24^ molecules which may be thought as subunits of salicylic acid. Comparing our results to these reports we find similar trends, as seen in Figure 2. For phenol, three π* resonances are reported whereas for formic acid, there is one clear π* resonance. For both phenol and formic acid, although expected, no  resonance is discernible in the cross section. As mentioned earlier, there are four resonant structures in the cross sections of salicylic acid, which can be related to the resonances of phenol and formic acid (although shifted in energy). To further investigate these resonances, we diagonalized the scattering Hamiltonian in the SEP configuration space, following the procedure described in the previous section. The resonant orbitals are shown in Figure 4 and their respective eigenvalues are listed in Table 2. It is interesting to compare the resonant orbitals for SA with those for its building blocks phenol^23^ and formic acid.^24^ Thus, we have performed electronic structure calculations, with a compact basis set, for both molecules to obtain the resonant orbitals, which are also shown in Figure 4. It is noted that the  resonant orbital of SA is mainly related to the  orbital from phenol,^23^ and the resonant energies are quite similar: while our calculations peak this resonance at 1.27 eV, somewhat higher than the measurements at 1.15 and 1.11 eV,^7,8^ for phenol this resonance was measured at 1.01 eV.^25^ On the other hand,  and  resonant orbitals of SA seem to be a superposition of the  from phenol with strong contributions in the carboxylic group. It turns out that, depending on the phase of the contributions, the resonance is stabilized and or destabilized: whereas the  resonance for phenol was calculated at 1.33 eV, our calculations peak the  and  resonances of SA at 0.023 and 3.60 eV, respectively. The  resonance of SA is strongly related to the  resonance of phenol, although some minor contribution of the carboxylic group is noted. When our calculated resonance positions are compared with the available measurements it is noted that whereas the second resonance is in very good agreement with the measurements, the first one is overstabilized and the third and fourth resonances are overestimated. This is mainly due to the difficulties in describing four π* resonances in a single symmetry. In Table 2 we summarize the resonance peak energies for SA.

**Table 2:** Comparison of the Resonant Peak Energies for SA with Previous ETS Measurements of Scheer et al.^7^ and Pshenichnyuk and Modelli^8^^a^

Orbital	SE	SEP	Eigenvalues *H*_*N*+1_	ETS^7^	ETS^8^
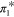	1.9	0.023	0.073	0.17	0.2
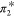	4.3	1.27	1.28	1.15	1.11
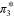	5.6	3.60	3.51	2.39	2.44
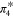	10.2	6.80	6.09	4.4	4.5

a All units are in eV.

It is well-known that the higher lying π* resonance in benzene and its derivatives is a mixture of shape and core-excited resonances. This was investigated in pyrazine by Winstead and McKoy through electronic structure and scattering calculations.^21^ Here we carried out additional electronic structure calculations on SA, using GAMESS,^12^ to investigate the nature of the higher lying  resonance. Following the same procedure adopted by Winstead and McKoy,^21^ we performed CISD calculations on the anion using the 6-311+G(1*d*, 1*p*) basis set at the ground state geometry of the neutral molecule. This geometry was optimized at the HF level using this same basis set. The active space in the CISD calculations included 6 active doubly occupied molecular orbitals, 1 singly occupied molecular orbital (SOMO) of *a*″ symmetry and 3 unoccupied molecular orbitals also of *a*″ symmetry. We computed the energies for the 4 lowest anion states of the *A*″ symmetry. The ground state, and the first and second excited states of the anion, which correspond to the resonances  to , have major contribution from configurations typical of a shape resonance. For instance, the ground and the first excited states have major contribution (with coefficient of 0.93) from configurations corresponding to the extra electron in the SOMO and in the lowest unoccupied molecular orbital (LUMO), respectively. In the case of the second excited state the major contribution is from a configuration corresponding to the extra electron in the LUMO+1 (coefficient 0.76). However, the third excited state of the anion, which corresponds to the  resonance, has major contribution from configurations that consider the excitation of one electron from one of the doubly occupied orbitals (coefficients of 0.53 and −0.47) and a small contribution from a shape-resonance configuration (coefficient of 0.30). The results of the CISD calculations support the mixed character of the  of shape and core-excited shape resonances. We also investigated the π* resonances of phenol looking at the ground, first and second excited states of the anion with calculations at the CISD level. These calculations were similar to the calculations of SA and showed that the ground state and the first excited state, which are related to the  and  resonances of phenol, have a major contribution (coefficient of 0.96) from configurations of a shape resonance, while the second excited state, which is related to the higher-lying  resonance of phenol, has contributions from configurations of shape and core-excited resonances. We can then relate the higher-lying  mixed resonance of SA to the higher-lying  mixed resonance of phenol.

Pshenichnyuk and Modelli^8^ reported the DEA spectrum of salicylic acid. In the low-energy regime (below around 4.0 eV) the main dissociation channels are the formation of [M-H]^−^ and [M-H_2_]^−^ species around 1 eV. The  resonance was assigned as responsible for the formation of these fragments due to its energy proximity with the fragment maximum in the DEA spectrum. According to the authors, the dehydrogenated molecular anion could be formed either by an indirect dissociation pathway, associated with the coupling between a π* and a σ* resonances through an out-of-plane bending, leading to the breaking of the O–H bond, or via a direct dissociation pathway associated with the direct formation of a σ* resonance. Although the absence of a clear σ* resonance signature in our calculations tends to point out that the former is the most likely dissociation pathway, one should note that for formic acid, the direct mechanism is favored.^26^ Thus, to assess which mechanism is responsible for the loss of an H atom, more detailed calculations along the reactive coordinate would be necessary.

The two hydrogen atoms associated with the [M-H_2_]^−^ species formed around 1 eV were theorized to come from the carboxylic and OH groups.^8^ Due to its proximity in energy, the  resonance was assigned as responsible for the formation of this fragment. The resonant orbital obtained in our work ( in Figure 4) does not present any component on the carboxylic group. Thus, our results do not corroborate the interpretation of Pshenichnyuk and Modelli,^8^ where the two hydrogens would come from O–H bonds. Instead, the resonant orbital presented in Figure 4 suggests that the two hydrogen atoms would come from the C–H bonds in the benzene ring of SA. Nevertheless, we note that these dissociation pathways need to be comprehensively probed in order to provide definitive answers to their nature, either by studying potential energy curves for the anions,^27^ or by experiments with deuterium substitutes.^28^

**Figure 5:**
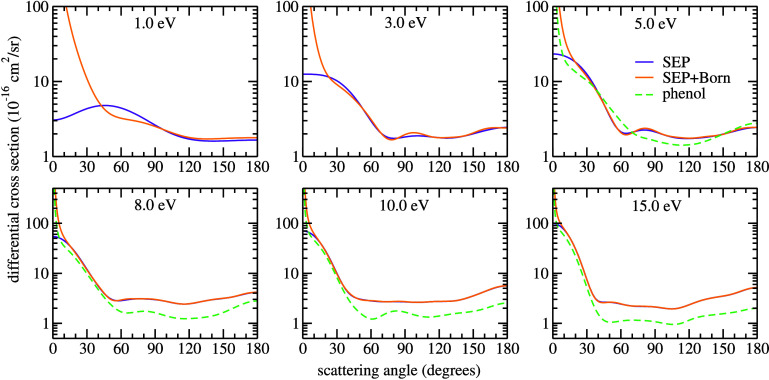
Differential cross sections for electron elastic scattering by salicylic acid. The curves are represented by different line styles and colors: the solid orange line corresponds to the SEP+Born approximation; the solid violet line represents the SEP approximation. The results from da Costa et al.^22^ for phenol are also shown in the dashed green line. We note that the results from da Costa et al.^22^ were calculated with different levels of multichannel coupling: 3ch-SEP, 13ch-SEP, 21ch-SEP, and 23ch-SEP, at scattering energies 5.0, 8.0, 10.0, and 15.0 eV, respectively.

The DCSs in the SEP and SEP+Born approximations are shown in Figure 5, alongside the results for phenol from da Costa et al.^22^ As expected, when comparing the present results obtained with and without the Born-closure procedure, the inclusion of the long-ranged electron-molecule interaction affects primarily the magnitude of the forward scattering. The qualitative comparison with the DCSs for phenol is good, especially for higher impact energies, as can be seen at 15 eV where the minima seen in the DCS for salicylic acid coincide with the ones seen in phenol. Quantitatively the cross sections for salicylic acid are higher than the ones for phenol over almost all scattering angles. Although this could be explained by the different molecular sizes, we note that the calculations performed by da Costa et al.^22^ take into account the multichannel coupling effect. This effect considers the flux competition between the elastic and inelastic channels, which is responsible for lowering the magnitude of the (elastic and inelastic) cross sections. Thus, the difference in magnitude could be related to the level of approximation in which the calculations were performed, and a direct quantitative comparison would be unfair.

## Conclusion

The study of electron interaction with molecules is key for understanding fundamental biological processes, as it sheds light on how low-energy electrons interact with biomolecules, influencing DNA damage, for example. In this work, we have presented calculated cross sections for low-energy electron elastic scattering by salicylic acid, an important biomolecule present in a class of drugs with analgesic properties. The elastic ICS, DCS, and MTCS were calculated with the SMC method within the SE and SEP approximations up to 15 eV. From our calculations we were able to characterize four π* resonances, located at around 0.023, 1.27, 3.60, and 6.80 eV, in the SEP approximation. While the latter is a mixed shape and core-excited resonance, the first three are shape resonances. A direct comparison was found between these four resonances of salicylic acid and the resonances for SA building blocks, phenol^23^ and formic acid.^24^ We also compared our results with experimental ETS data,^7,8^ revealing a good agreement in the positions of the two lowest calculated resonances, while the higher-lying resonances tend to overestimate the corresponding experimental positions. With the aim of shedding some light in the DEA mechanisms for SA, we have also discussed the role of the second π* resonance in the production of [M-H]^−^ and [M-H_2_]^−^ species, in a close comparison with available measurements.^8^ We have also found some evidence of the role played by electronic exctitations in the low-energy electron dynamics with salicylic acid. This issue should be investigated, both theoretically and experimentally, in future works. Moreover, we hope that this work pave the way for future research regarding electron interactions with salicylic acid.
